# Biochemical and Antiparasitic Properties of Inhibitors of the Plasmodium falciparum Calcium-Dependent Protein Kinase PfCDPK1

**DOI:** 10.1128/AAC.02959-14

**Published:** 2014-10

**Authors:** Keith H. Ansell, Hayley M. Jones, David Whalley, Alisdair Hearn, Debra L. Taylor, Emmanuel C. Patin, Timothy M. Chapman, Simon A. Osborne, Claire Wallace, Kristian Birchall, Jonathan Large, Nathalie Bouloc, Ela Smiljanic-Hurley, Barbara Clough, Robert W. Moon, Judith L. Green, Anthony A. Holder

**Affiliations:** a MRC Technology, Centre for Therapeutics Discovery, Mill Hill, London, United Kingdom; b Division of Parasitology, MRC National Institute for Medical Research, Mill Hill, London, United Kingdom

## Abstract

PfCDPK1 is a Plasmodium falciparum calcium-dependent protein kinase, which has been identified as a potential target for novel antimalarial chemotherapeutics. In order to further investigate the role of PfCDPK1, we established a high-throughput *in vitro* biochemical assay and used it to screen a library of over 35,000 small molecules. Five chemical series of inhibitors were initially identified from the screen, from which series 1 and 2 were selected for chemical optimization. Indicative of their mechanism of action, enzyme inhibition by these compounds was found to be sensitive to both the ATP concentration and substitution of the amino acid residue present at the “gatekeeper” position at the ATP-binding site of the enzyme. Medicinal chemistry efforts led to a series of PfCDPK1 inhibitors with 50% inhibitory concentrations (IC_50_s) below 10 nM against PfCDPK1 in a biochemical assay and 50% effective concentrations (EC_50_s) less than 100 nM for inhibition of parasite growth *in vitro*. Potent inhibition was combined with acceptable absorption, distribution, metabolism, excretion, and toxicity (ADMET) properties and equipotent inhibition of Plasmodium vivax CDPK1. However, we were unable to correlate biochemical inhibition with parasite growth inhibition for this series overall. Inhibition of Plasmodium berghei CDPK1 correlated well with PfCDPK1 inhibition, enabling progression of a set of compounds to *in vivo* evaluation in the P. berghei rodent model for malaria. These chemical series have potential for further development as inhibitors of CDPK1.

## INTRODUCTION

Malaria is caused by infection with parasitic protozoa of the genus Plasmodium. There are five Plasmodium species that cause human infection, of which the most important is Plasmodium falciparum. Between 1980 and 2010, malaria was responsible for more than 1 million deaths and over 200 million clinical cases annually across the globe (1). The development of resistance to antimalarial drugs by the parasite has focused attention on the need for new effective therapies. Although artemisinin combination therapy (ACT) has been widely deployed and shown to be very effective, clinical resistance to artemisinin appears to have developed in southeastern Asia (2, 3). Therefore, compounds against new molecular targets that are orally active and effective against all human malaria parasites are urgently required.

Protein kinase inhibitors have been developed for a range of medical applications, and several have progressed to the clinic, implicating protein kinases as a class of target with great therapeutic potential (4). In this context, the malaria parasite kinome (the set of protein kinases encoded in the genome of the malaria parasite) has been investigated to identify novel prospective therapeutic targets (5, 6). One potential group is the calcium-dependent protein kinases (CDPKs), a family of kinases not found in mammals. CDPKs are found only in higher plants, algae, and alveolates, a group that includes the Apicomplexa, the family of parasitic protozoa that cause diseases of great clinical and economic importance in humans and animals, including malaria (7). CDPKs contain a kinase domain and a calmodulin-like, calcium-binding regulatory domain (8). The P. falciparum genome contains five genes encoding canonical CDPKs, and they have been implicated in a range of biological processes at different stages of the parasite life cycle (9). The fact that these enzymes are absent from the vertebrate hosts of these parasites suggests that they may represent useful targets for the development of antimicrobial agents.

The stage of the parasite life cycle responsible for disease is the asexual blood stage, a cyclic process in which the parasite invades and then develops and multiplies within a red blood cell, progressing through the so-called ring, trophozoite, and schizont stages. Following nuclear and cell division that occurs at the schizont stage, newly formed merozoites are released from the infected cell, and these merozoites bind to and invade new red blood cells. In the case of P. falciparum, this cycle takes approximately 48 h and produces 20 to 30 new merozoites. Nuclear division and cell division are accompanied by formation of the parasite's pellicle comprised of the plasma membrane (PM) and the inner membrane complex (IMC) that lies just below the PM (10). The IMC consists of flattened membranous sacs or alveoli that characterize these protozoa. The space between the PM and IMC is the location of the actomyosin motor that drives merozoite invasion of red blood cells. Myosin A is linked to the IMC by virtue of its association with a complex of membrane-anchored proteins, including the 45- and 50-kDa glideosome-associated proteins (GAP45 and GAP50) and a myosin light chain called myosin A tail domain-interacting protein (MTIP) (11). CDPK1 is also located between the IMC and PM and is anchored to the PM by dual acylation of N-terminal residues (12). P. falciparum calcium-dependent protein kinase 1 (PfCDPK1) has been shown to phosphorylate MTIP and GAP45 *in vitro*, although it remains to be established that these proteins are its principal substrates *in vivo* (13).

CDPK1 has been validated as a potential drug target by both genetic and chemical biology approaches. Initial genetic studies in which unsuccessful attempts were made to disrupt the *cdpk1* gene in both P. falciparum and the rodent parasite Plasmodium berghei suggested that the enzyme is essential for growth at the asexual blood stage (5, 14). More recently, conditional expression of the regulatory domain, which interacts with the enzyme to inhibit it, was shown to inhibit growth of the parasite at the early schizont stage (15). Earlier inhibitor studies have also targeted CDPK1. In one study, a high-throughput screen (HTS) resulted in the identification of purfalcamine, a CDPK1 inhibitor that inhibited parasite egress (merozoite release) at the end of schizogony (14). In a second study, a series of inhibitors of the enzyme was developed, but their effect on parasite growth was not tested (16). Together, these genetic and inhibitor studies suggest that CDPK1 might be a good target for drug development to inhibit the parasite growth and multiplication that is responsible for the disease.

In this study, we developed a HTS based on PfCDPK1 phosphorylation of MTIP. Several classes of hit compounds were identified and characterized and used as the basis for the synthesis of more-active compounds. The interaction of these compounds with the enzyme was investigated in detail, and the ability of some to inhibit parasite growth *in vitro* was examined.

## MATERIALS AND METHODS

### Expression and purification of recombinant enzymes.

The Plasmodium falciparum *cdpk1* gene (*Pfcdpk1*) was amplified from 3D7 schizont cDNA using primers 5′-CGGGATCCATGGGGTGTTCACAAAGTTC-3′ and 5′-CGCTCGAGTCATTATGAAGATTTATTATCACAA-3′ and cloned into the BamHI and XhoI sites of pGEX6P1 (GE Healthcare).

Gatekeeper mutants were generated by site-directed mutagenesis using the QuikChange II kit (Stratagene) with pGEX6P1-PfCDPK1 (PfCDPK1 is the Plasmodium falciparum calcium-dependent protein kinase 1) as a template and primers. For T145Q, the primer 5′-TTTTATTTAGTA**CAA**GAATTTTATGAAGGTGGGGA-3′ and its reverse complement were used, while for T145G, the primer 5′-TTTTATTTAGTA**GGC**GAATTTTATGAAGGTGGGGA-3′ and its reverse complement were used (the altered codons are shown in boldface type in both cases).

Synthetic genes encoding Plasmodium vivax CDPK1 (PvCDPK1) and Plasmodium berghei CDPK1 (PbCDPK1) (Geneart) were also cloned into the BamHI and XhoI sites of pGEX6P1.

After transformation into Escherichia coli BL21 Gold cells (Stratagene), cultures grown in Terrific broth were treated with 1 mM isopropyl-β-d-1-thiogalactopyranoside (IPTG) overnight at 18°C to induce protein expression. The cell pellet was resuspended in 10 ml/g lysis buffer [50 mM Tris-HCl (pH 8.8), 250 mM NaCl, 20 mM KCl, 5 mM MgCl_2_, 1 mM Tris(2-carboxyethyl)phosphine (TCEP), 5% glycerol, 1× complete protease inhibitors (Roche), 2 mg/ml lysozyme (Sigma-Aldrich), and 1 μl/ml benzonase (Roche)] and incubated on a roller mixer overnight at 4°C. Insoluble material was removed by centrifugation at 40,000 × *g*. Glutathione Sepharose 4b beads (GE Healthcare) were added to the supernatant and incubated, with mixing, for 2 h at 4°C. The beads were packed into a glass Econo-column (Bio-Rad) and washed first with 50 volumes of wash buffer 1 (20 mM Tris-HCl [pH 8.8], 250 mM NaCl, 2 mM EDTA [pH 8.0], 0.5 mM TCEP) and then with 20 volumes of wash buffer 2 (20 mM Tris-HCl [pH 8.8], 100 mM NaCl, 0.5 mM TCEP). The beads were then resuspended in 5 volumes of wash buffer 2 containing 2 μg/ml PreScission Protease (GE Healthcare). The sample was incubated, with mixing, overnight at 4°C. The beads were placed in a glass Econo-column (Bio-Rad), and the solution containing CDPK1 was collected by gravity flow. The sample was concentrated using a Vivaspin 20 concentrator with a 10-kDa-molecular-size cutoff (Vivascience) and loaded onto a Sephadex S200 gel filtration column (GE Healthcare). Fractions containing CDPK1 were identified and concentrated as described before. Protein concentrations were determined by measuring *A*_280_ of samples.

Catalytically inactive PfCDPK1 D191N (PfCDPK1 with the D191N substitution) and myosin A tail domain-interacting protein (MTIP) were expressed and purified as previously described (13).

### Compound libraries.

The LOPAC 1280 (LOPAC stands for library of pharmacologically active compounds) collection of 1,280 pharmacologically active compounds was purchased from Sigma-Aldrich. A diverse collection of ∼26,000 compounds was selected from various commercial suppliers by applying “lead-like” filters *in silico* (e.g., logarithm of a compound's partition coefficient between *n*-octanol and water [cLogP] of 0 to 4, polar surface area [PSA] of <120, H-bond donors of <3, H-bond acceptors of <6, freely rotatable bonds of <6). A kinase-focused set of 8,100 compounds was purchased from Biofocus and ChemDiv.

### High-throughput screening and biochemical assays.

A high-throughput screen (HTS) of 35,422 compounds comprising a diverse set of small molecules from a variety of commercial suppliers and 8,100 kinase-focused compounds was performed using Kinase-Glo Plus (Promega) to measure ATP depletion resulting from the kinase reaction. Compounds (final concentration of 10 μM) were added to 22-μl reaction mixtures in white 384-well plates containing 50 nM full-length recombinant PfCDPK1 and 8 μM MTIP in assay buffer 1 (Tris-HCl buffer [pH 8.0] containing 0.1 mM EGTA, 0.2 mM CaCl_2_, 1 mM dithiothreitol [DTT], and 0.01% Triton X-100) and incubated for 30 min at room temperature prior to initiating the reaction with final concentrations of 10 μM ATP (*K_m_*) and 20 mM MgCl_2_. The reactions were allowed to proceed for 60 min at ambient temperature and stopped by the addition of 22 μl Kinase-Glo Plus detection reagent. Luminescence proportional to the remaining ATP at the end of the reaction was measured using a Pherastar plate reader (BMG Labtech). The raw luminescence data were normalized to percentage activity of controls where percent activity = (relative luminescence units [RLU] in the presence of test compound − mean RLU of the low controls)/(mean RLU of the high controls − mean RLU of the low controls) × 100. High controls consisted of complete reaction mixtures plus vehicle (1% dimethyl sulfoxide [DMSO]), and low controls contained all components except enzyme. “Hits” were identified as compounds that gave <50% residual activity in the primary screen. Unless otherwise stated, 50% inhibitory concentration (IC_50_) determinations were performed in the Kinase-Glo Plus assay in the same way except that the enzyme concentration was reduced to 10 nM and the incubation time was increased to 120 min. Ten-point dose-response curves were obtained from half-log dilutions of test compounds diluted in assay buffer 1 at a constant final DMSO concentration of 1% and incubated for 30 min with the enzyme prior to initiation of the kinase reaction with 10 μM ATP and 20 mM MgCl_2_. IC_50_s were determined by four-parameter logistical fitting of the data using XL-fit software (ID Business Solutions [IDBS]). pIC_50_ values were calculated using the relationship pIC_50_ = −log_10_ IC_50_.

Alternatively, IC_50_s were determined in kinetic mode using a rhodamine-labeled ParM (Rh-ParM) ADP sensor (17). For these experiments, 5 μl of P. falciparum (wild type [WT]), P. berghei, or P. vivax CDPK1, or P. falciparum gatekeeper mutants were diluted in assay buffer 2 (Tris-HCl buffer [pH 8.0] containing 1 mM CaCl_2_, 1 mM DTT, 25 mM KCl, 100 μM EGTA, and 0.01% [vol/vol] Triton X-100) to a final concentration of 10 nM and mixed with 10 μl Rh-ParM at a final concentration of 100 nM in black 384-well plates (Corning). Compounds were diluted in half-log series in DMSO, and 2-μl volumes of diluted compound, or vehicle to high and low controls, were added to the enzyme and incubated for 30 min at room temperature before initiation of the reaction with 5 μl of 20 mM MgCl_2_ and ATP at the appropriate previously determined *K_m_* value. Alternatively, for mechanism-of-action experiments, the assay was performed in the same way except that ATP was added to a final concentration of 30 μM and in parallel to 1 mM. Fluorescence measurements (excitation wavelength [λ_ex_] of 545 nm and emission wavelength [λ_em_] of 590 nm) were made at 300-s time points using a Pherastar Plus plate reader (BMG Labtech), and an appropriate endpoint was selected in the linear phase of the reaction. The data were normalized to percentage activity and plotted to obtain the IC_50_ as described above. IC_50_s were calculated from a four-parameter logistical fit of the data using the XL-fit software.

For determination of PfCDPK1 ATPase activity, enzymatic release of P_i_ was measured in real time using a rhodamine-labeled phosphate-binding protein (Rh-PBP) biosensor (18). Twenty-microliter reaction mixtures containing final concentrations of 20 nM PfCDPK1 and 10 μM Rh-PBP diluted in assay buffer 1 were initiated with 100 μM ATP and 20 mM MgCl_2_ in black 384-well plates (Corning). Fluorescence measurements (λ_ex_ = 545 nm; λ_em_ = 590 nm) were made at 300-s time intervals using a Pherastar Plus plate reader (BMG Labtech). Background fluorescence in the absence of enzyme was subtracted from the signal, and phosphate release was quantified with reference to a standard P_i_ curve.

Radiometric determination of PfCDPK1 activity was measured by enzymatic incorporation of γ-^33^P into MTIP. Reaction mixtures (20-μl final volume) containing 10 nM PfCDPK1 and 20 μM MTIP in assay buffer 1 were preincubated with various concentrations of test compounds or DMSO/vehicle for 30 min in U-bottomed polypropylene 96-well plates (Matrix). The reactions were initiated with a final concentration of 10 μM ATP containing 0.2 μCi of [γ-^33^P]ATP and 20 mM MgCl_2_. The reaction mixtures were incubated for 1 h at room temperature (RT). Then the reactions were terminated with 20 μl of 50% (vol/vol) orthophosphoric acid. The reaction products were captured on glass-filter capture plates (Unifilter-96 GF/C; PerkinElmer) with an automated cell harvester (Harvester96; Tomtec). After 10 washes with 200 μl of 20% (vol/vol) orthophosphoric acid and 1 wash with 100% ethanol, the plates were dried and sealed before the addition of 25 μl of Microscint 20 (PerkinElmer) to all wells and counted in a TopCount NXT (PerkinElmer). IC_50_s were determined by four-parameter logistical fit of the data using GraphPad Prism software (GraphPad Software, Inc.).

### Enzyme kinetic measurements.

Kinetic parameters for PfCDPK1 were determined using Kinase-Glo Max (Promega), which is linear to a maximum concentration of 500 μM ATP. The assays were performed as described above, except that the ATP concentration was varied in the presence of 20 μM MTIP to obtain the *K_m_* for ATP, or alternatively, the ATP concentration was fixed at 100 μM and the MTIP concentration was varied to obtain the *K_m_* for MTIP. The reactions were terminated with the Kinase-Glo Max reagent after 10 min to obtain the initial reaction velocity, and the data were processed using a nonlinear fit to the Michaelis-Menten equation using GraphPad Prism software. Alternatively, for PvCDPK1, PbCDPK1, and PfCDPK1 gatekeeper mutants, kinetic parameters were obtained using the Rh-ParM ADP sensor assay (17). ATP *K_m_* values were obtained from initial velocity measurements made in the presence of various concentrations of ATP and a final concentration of Rh-ParM ADP of 100 nM in the presence or absence of 8 μM MTIP. Initial velocity data were fitted to the Michaelis-Menten equation with GraphPad Prism software.

### Thermal shift assay.

Purified recombinant PfCDPK1 was diluted to 1 μM in 10 mM HEPES buffer (pH 7.5) containing 150 mM NaCl, 1 mM Ca^2+^, and 1/1,000 SYPRO orange dye (Invitrogen). Test compounds were prediluted to 400 μM in 40% (vol/vol) DMSO in water, and 1 μl of diluted compound was added to 39 μl of enzyme/dye mix in white 96-well quantitative PCR plates (Thermo Scientific) to give a final compound concentration of 10 μM. Reference thermal melting temperatures (*T_m_*s) were obtained in parallel by the addition of 1 μl of 40% (vol/vol) DMSO to 39 μl of diluted PfCDPK1. The plates were sealed with transparent adhesive covers (Bio-Rad) and subjected to a temperature gradient from 25 to 95 K at a rate of 1 K/min using a quantitative PCR machine (MX3005P; Stratagene). Fluorescence data were acquired at 1-min intervals using the 6-carboxyfluorescein (FAM)/5-carboxy-X-rhodamine (ROX) filter set (λ_ex_ = 492 nm; λ_em_ = 610 nm), and the raw data were exported to Excel (Microsoft) for analysis. Data were processed to identify the fluorescence maxima and minima, and the midpoints of melting curves were determined by fitting to the Boltzmann equation using XL-fit software (IDBS). Results were expressed as Δ*T_m_* values relative to DMSO controls where Δ*T_m_* = *T_m_* in the presence of inhibitor − *T_m_* of the DMSO controls.

### FACS assay for parasite growth.

Fifty percent effective concentrations (EC_50_s) for small-molecule inhibition of parasite growth *in vitro* were obtained by quantification of red blood cell infection by fluorescence staining of DNA and fluorescence-activated cell sorting (FACS) analysis. Synchronized P. falciparum 3D7 cultures were established, and schizont-stage cells were used to infect human erythrocytes. After 24 h, cultures were diluted to 1% parasitemia while maintaining 2% hematocrit. Test compounds and controls were prepared by serial 2-fold dilution series in 1% DMSO and added in duplicate to 100-μl parasite cultures (0.05% DMSO final concentration in all culture samples) in 96-well plates. DMSO was added to each of 8 wells containing uninfected erythrocytes (low controls) and 8 wells containing parasitized erythrocytes (high controls) per 96-well plate. Standard titrations of pyrimethamine and artemisinin were included in each experiment. After a further 48 h, 40 μl from each well was transferred into the wells on fresh 96-well plates. Ten microliters of 50 μg/ml dihydroethidine (Sigma-Aldrich) diluted in phosphate-buffered saline (PBS) was added to each well, mixed, and incubated for 20 min at 37°C. The cells were fixed by the addition of glutaraldehyde, diluted in PBS, to a final concentration of 0.04%. The plates were incubated at 4°C for 1 h, 40 μl of supernatant was removed from each well, discarded, and replaced with 40 μl of PBS. Data were acquired using a Guava easyCyte Plus flow cytometer with ExpressPro software. EC_50_s were obtained by four-parameter logistical fit of the data using GraphPad Prism software. pEC_50_ values were calculated using the relationship pEC_50_ = −log_10_ EC_50_. Pyrimethamine and artemisinin were included in each experiment as positive controls and gave mean EC_50_s of 8 nM and 13 nM, respectively.

## RESULTS

### Development of a high-throughput screening assay for the identification of novel PfCDPK1 inhibitors.

**FIG 1:**
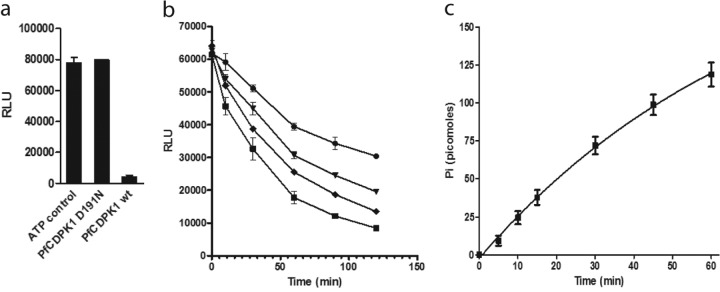
(a) ATP depletion in the presence of wild-type (wt) PfCDPK1 or D191N mutant in the absence of an additional substrate. Reaction mixtures contained 2.6 μM enzyme and 100 μM ATP or ATP alone (control), and the reactions were allowed to proceed for 45 min before detection of remaining ATP using the Kinase-Glo Plus reagent. Under these conditions, conversion of ATP by wt PfCDPK1 was approximately 95%. RLU, relative luminescence units. (b) ATP depletion in the presence of 50 nM wt PfCDPK1 and 0 μM (circles), 4 μM (triangles), 8 μM (diamonds), and 16 μM (squares) MTIP from an initial ATP concentration of 20 μM. Reactions were terminated with Kinase-Glo Plus reagent at various time intervals. (c) Kinetic plot showing enzymatic production of inorganic phosphate by PfCDPK1 in the presence of ATP alone. PfCDPK1 (20 nM [0.4 pmol]) was incubated with 100 μM ATP in the presence of 10 μM Rh-PBP. Raw fluorescence data were converted to the equivalent of phosphate with reference to a standard curve. Panels a to c show results representative at least three independent experiments (standard deviations [SD] indicated by the error bars).

**FIG 2:**
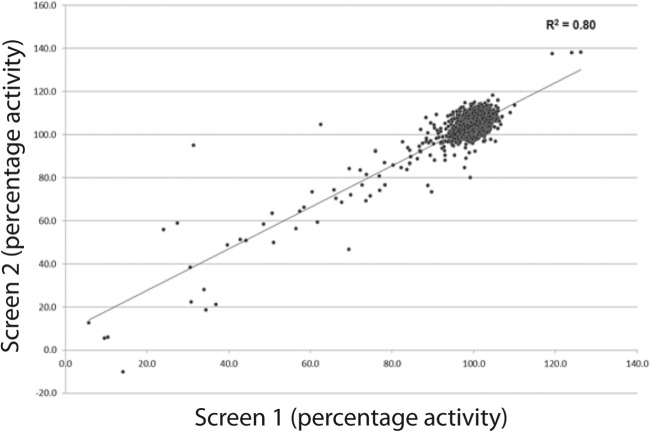
Experimental reproducibility for a screen of 1,280 compounds from the LOPAC 1280 library performed on two separate occasions. The mean *Z*′ statistics were 0.8 and 0.7 for screens 1 and 2, respectively, and 90% of the hits identified in screen 1 (threshold < 70% activity) were confirmed in screen 2.

In order to identify novel small-molecule inhibitors of PfCDPK1, we developed an *in vitro* high-throughput biochemical assay using recombinant PfCDPK1 enzyme and a previously identified physiological substrate, MTIP (13). To adapt the assay to a nonradiometric format that was suitable for high-throughput screening of small-molecule libraries, we chose to measure ATP remaining at the end of the reaction using a luminescence readout (Kinase-Glo Plus; Promega). Using this assay, the *K_m_* for ATP was determined to be approximately 10 μM in the presence of 20 μM MTIP, and the *K_m_* for MTIP was approximately 8 μM in the presence of 100 μM ATP. The *K_m_* for MTIP was comparable to that measured previously using an enzyme-coupled assay, while the ATP *K_m_* was approximately 10-fold lower in the Kinase-Glo Plus assay. Interestingly, we observed a high background turnover of ATP by wild-type PfCDPK1, but not by the catalytically inactive mutant PfCDPK1 D191N, in the absence of MTIP (Fig. 1a). An MTIP dose-dependent increase in the rate of ATP turnover was nevertheless apparent in the assay (Fig. 1b). Some conversion of ATP in the absence of a substrate was expected due to autophosphorylation, and a total of 10 sites have been identified previously (19, 20). However, the conversion of ATP in the absence of substrate was not stoichiometric with respect to the amount of enzyme present in the reaction mixture (more than 290 pmol of ATP consumed per pmol of enzyme) and could not be attributed to autophosphorylation alone, instead being consistent with an ATPase activity. To investigate this further, we assayed for inorganic phosphate (P_i_) production during the reaction in the absence of MTIP using a rhodamine-labeled phosphate biosensor (Rh-PBP). The Rh-PBP undergoes a conformational change on binding to P_i_, resulting in an increase in fluorescence at 580 nM. Thus, it provides a kinetic readout for P_i_ produced during an enzymatic reaction (18). The detection of P_i_ was consistent with PfCDPK1-mediated hydrolysis of ATP in the absence of MTIP (Fig. 1c), although the rate of ATP depletion was increased in its presence. Therefore, when selecting the conditions for high-throughput screening, MTIP was included in the reaction mixture. After optimization of the assay, including the ratio of signal to background under linear reaction conditions and DMSO tolerance, the final screening protocol was established in 384-well plate format with automated liquid handling. To determine the performance of the HTS assay, 1,280 compounds from the LOPAC 1280 set of pharmacologically active compounds were screened on two separate occasions (Fig. 2). The assay performance was robust, as shown by *Z*′ statistics of 0.8 and 0.7 on runs one and two, respectively (21). Appl**y**ing a threshold of greater than 30% inhibition, the hit confirmation rate was determined to be 90%. Therefore, the assay conditions were considered suitable for a wider screen of the in-house small-molecule libraries.

### Five chemical series of PfCDPK1 inhibitors were identified from the HTS.

PfCDPK1 was further screened against 35,422 compounds in total, including around 26,000 diversity set compounds and a kinase-focused set of 8,100 compounds. The mean *Z*′ statistic for the screen was 0.74, which was consistent with the performance obtained in the pilot screen. In total, 801 hits showing inhibition greater than 50% at 10 μM were confirmed by repeat, thereby giving an overall confirmed hit rate of 2.3%. Notably, the confirmed hit rate for the kinase-focused collection (4.2%) was more than 10-fold higher than that obtained from the general diversity set (0.4%), indicating that PfCDPK1 is susceptible to inhibition by the enriched kinase chemical templates present in this library. Indeed, a series of 2,6,9-trisubstituted purine inhibitors of PfCDPK1 had been identified previously from a screen of 20,000 kinase-directed templates (14).

**FIG 3:**
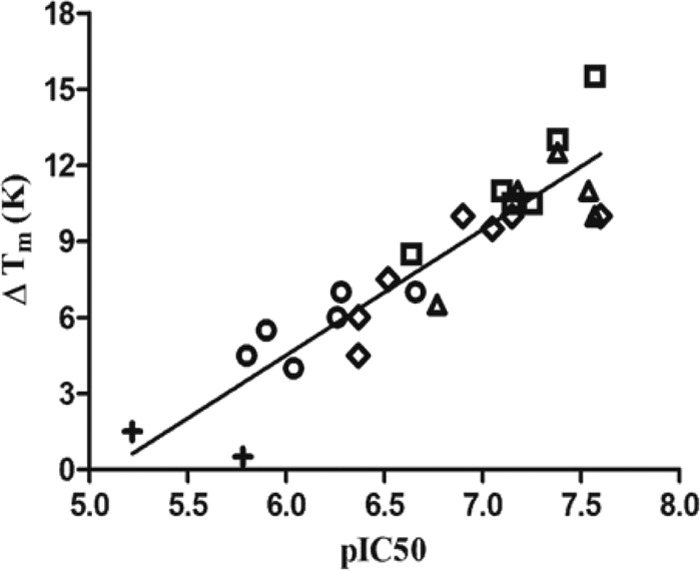
Relationship between pIC_50_ and thermal melting temperature induced by inhibitor binding to PfCDPK1. Data are for a panel of 26 PfCDPK1 inhibitors representing chemical series 1 to 5 (series 1 [triangles], series 2 [squares], series 3 [diamonds], series 4 [circles], and series 5 [plus signs]) (*r* = 0.91; *P* < 0.0001).

After removal of promiscuous hits and medicinal chemistry triage, 105 compounds from the confirmed hit list were short-listed for IC_50_ determinations. Dose-response curves were obtained for 101 compounds, including 66 compounds with an IC_50_ less than 5 μM, the most potent having an IC_50_ of 66 nM. From this set, five chemical series were identified for further study: imidazopyridazines (series 1), pyrazolopyrimidines (series 2), azabenzimidazoles (series 3), isoxazole amides (series 4), and pyrazinones (series 5) (Table 1). We tested examples from each of these series in a biophysical assay based on their ability to stabilize PfCDPK1 to denaturation by heat. Consistent with the observed biochemical inhibition, thermal shift data indicated stabilization of the enzyme of between 0.5 and 15.5 K in comparison to vehicle controls with significant correlation between Δ*T_m_* and IC_50_s obtained in the Kinase-Glo Plus assay across all series (*r* = 0.91; *P* < 0.0001) (Fig. 3).

**TABLE 1:**
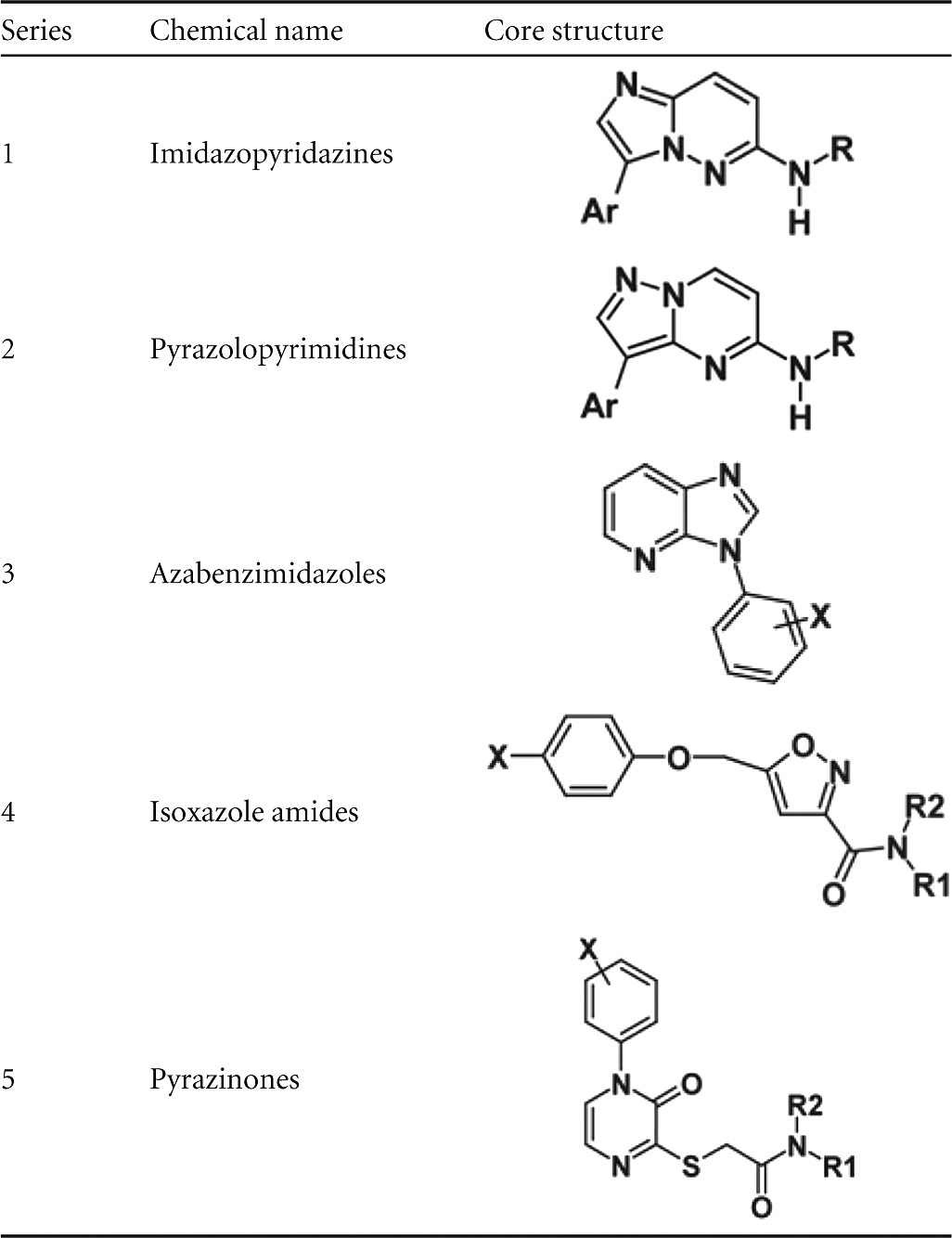
Five chemical series of PfCDPK1 inhibitors identified from a screen of 35,422 small molecules

### Mechanism of action.

**FIG 4:**
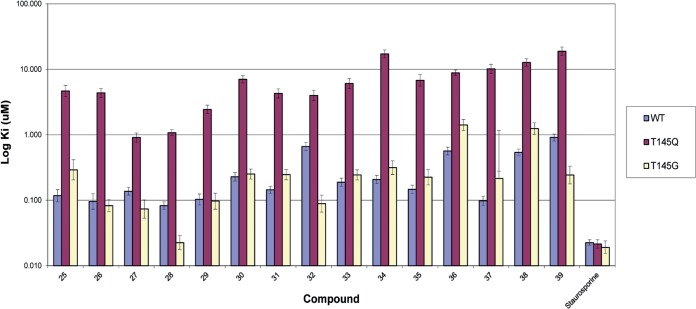
Effect of the ATP site gatekeeper residue on inhibitor affinity. *K_i_* values for a panel of compounds from series 1 and 2 and for staurosporine were determined in the absence of MTIP using the Rh-ParM ADP sensor (the 95% confidence limits indicated by the error bars).

To investigate the mechanism of action of these inhibitors, we determined IC_50_s for a panel of compounds from all chemical series in the presence of initial ATP concentrations of 30 μM and 1 mM. Exemplar compounds from each of the five chemical series showed reduced potency at the higher concentration of ATP, indicative of ATP competitive behavior (Table 2). Subsequently, series 1 (imidazopyridazines) and series 2 (pyrazolopyrimidines) were prioritized for chemical optimization based on promising initial structure-activity relationships (SARs), evidence of inhibition of parasite growth, and chemical tractability (the structures of all compounds studied further are provided in the supplemental material). Examples from these series were first tested in a radiometric assay in the presence of MTIP, and all were confirmed as inhibitors of the kinase activity (Table 3). The IC_50_s obtained in the radiometric assay were lower on average by a factor of 3.7-fold, presumably due to detection of the ATPase activity in the ATP depletion assay. Since the inhibitors were predicted to bind at the ATP site, we wanted to determine whether their potency would be influenced by the amino acid residue present at the “gatekeeper” position (position 145 in PfCDPK1). In the wild-type PfCDPK1 enzyme, the gatekeeper residue is threonine, a relatively small amino acid. Substitution of threonine with an amino acid possessing a bulkier side chain might have a differential effect on inhibitor binding compared with ATP binding. This approach has been used previously to show on-target specificity for inhibitors of the malarial cGMP-dependent kinase PfPKG (P. falciparum cGMP-dependent kinase), which also has a threonine gatekeeper in the wild-type enzyme (22). Therefore, we expressed a PfCDPK1 mutant containing glutamine at the gatekeeper position (T145Q) and another with the smallest amino acid glycine as a conservative change (T145G). The mutants were found to be enzymatically active with ATP *K_m_* values of 45 μM and 56 μM for T145Q and 86 μM and 98 μM for T145G in the presence or absence of 8 μM MTIP, respectively. Next we derived *K_i_* values for a panel of compounds representing series 1 and 2, using the Cheng-Prussov equation for competitive inhibition (Fig. 4). We found that all of the compounds were sensitive to the T145Q mutation, with *K_i_* values increasing by 6- to 103-fold compared to the WT enzyme. In contrast, there were no substantial differences in inhibitor potency between the wild-type gatekeeper and the enzyme with a glycine at this position or for staurosporine between the wild-type enzyme and enzyme with the T145Q mutation. Thus, a reduction in potency against the PfCDPK1 T145Q mutant enzyme was deemed indicative of on-target binding at the ATP site for further inhibitors synthesized during the hit to lead program.

**TABLE 2:** Analysis of the effect of the ATP concentration on inhibitor potency

Compound	Series	IC_50_ (nM)^*a*^		IC_50_ ratio (IC_50_ with 1 mM ATP/IC_50_ with 30 μM ATP)
		With 1 mM ATP	With 30 μM ATP	
10	1	1,698 (405)	64 (7)	27
11	1	1,375 (382)	49 (9)	28
12	1	3,908 (1,627)	107 (25)	37
13	2	3,583 (537)	212 (57)	17
14	2	745 (178)	28 (6)	27
15	2	270 (17)	23 (2)	12
16	3	2,974 (64)	77 (4)	39
17	3	1,376 (256)	51 (4)	27
18	3	4,533 (269)	86 (8)	53
19	4	24,176 (4,945)	72 (12)	336
20	4	31,678 (11,479)	728 (56)	44
21	4	5,933 (2,059)	143 (34)	41
22	5	>100,000	34,202 (7,136)	>3
23	5	>100,000	39,766 (10,371)	>3
24	5	>100,000	31,164 (3,147)	>3
Staurosporine	n/a^*b*^	161 (49)	11 (1)	15

a Mean IC_50_s were determined using ADP detection (Rh-ParM) in the absence of MTIP (*n* = 2) (standard deviations shown in parentheses).

b n/a, not applicable.

**TABLE 3:** Confirmation of HTS hits by radiometric assay

Compound	Series	IC_50_ (nM)^*a*^		IC_50_ ratio (ATP depletion/radiometric assay)
		ATP depletion	Radiometric assay	
10	1	65	24	2.7
11	1	65	22	3.0
12	1	200	34	5.9
14	2	52	15	3.5
15	2	32	12	2.7
47	1	27	7	3.9
50	1	41	10	4.1

a IC_50_s were determined in the Kinase-Glo Plus (ATP depletion) and radiometric assays in the presence of ATP and MTIP at their respective *K_m_* values.

### Relationship between biochemical inhibitor potency and inhibition of parasite growth *in vitro*.

**FIG 5:**
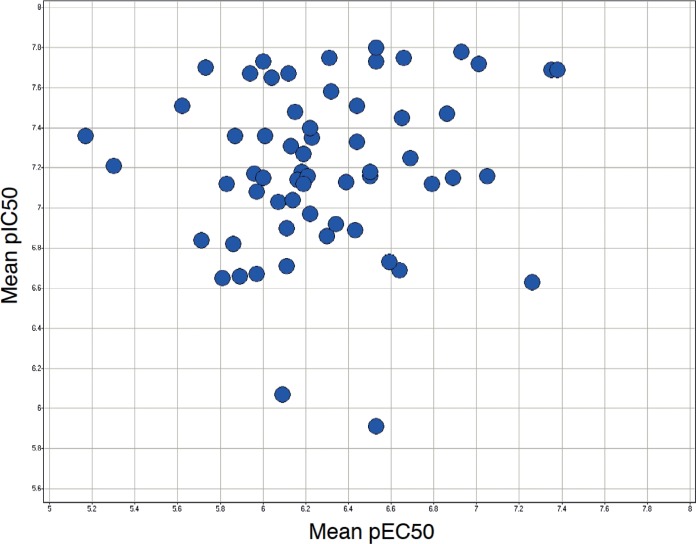
Relationship between inhibitor potency against PfCDPK1 and parasite growth inhibition. A total of 61 imidazopyridazine compounds (series 1) with mean pIC_50_ ranging from 5.9 to 7.8 and pEC_50_ ranging from 5.2 to 7.4 (*r* = 0.13; *P* = 0.30) are shown. Data correspond to a mean of at least two independent biochemical and parasite growth assays.

To determine the effects of the PfCDPK1 inhibitors on parasite growth, an *in vitro* fluorescence-based FACS assay was used, whereby parasite-infected red blood cells were quantified using a DNA-sensitive dye. For these experiments, synchronized P. falciparum 3D7 cultures were established, and 24 h postinvasion, the cultures were treated with PfCDPK1 inhibitors. After one further round of parasite replication (72 h after initial invasion), cultures were incubated with dihydroethidine and fixed prior to determination of parasitemia by FACS analysis. EC_50_s were calculated based on the percent parasitemia in the presence of a range of PfCDPK1 inhibitor concentrations relative to vehicle controls. Following medicinal chemistry efforts based primarily on the optimization of series 1, we identified a number of compounds that were potent inhibitors of both isolated enzyme and parasite growth *in vitro* (pIC_50_ > 8 and pEC_50_ > 7) (23, 24). However, we did not find any overall correlation between the observed inhibition of parasite growth and the biochemical potency of the compounds against the isolated enzyme within the series (Fig. 5).

### Cross-species activities of PfCDPK1 inhibitors.

**FIG 6:**
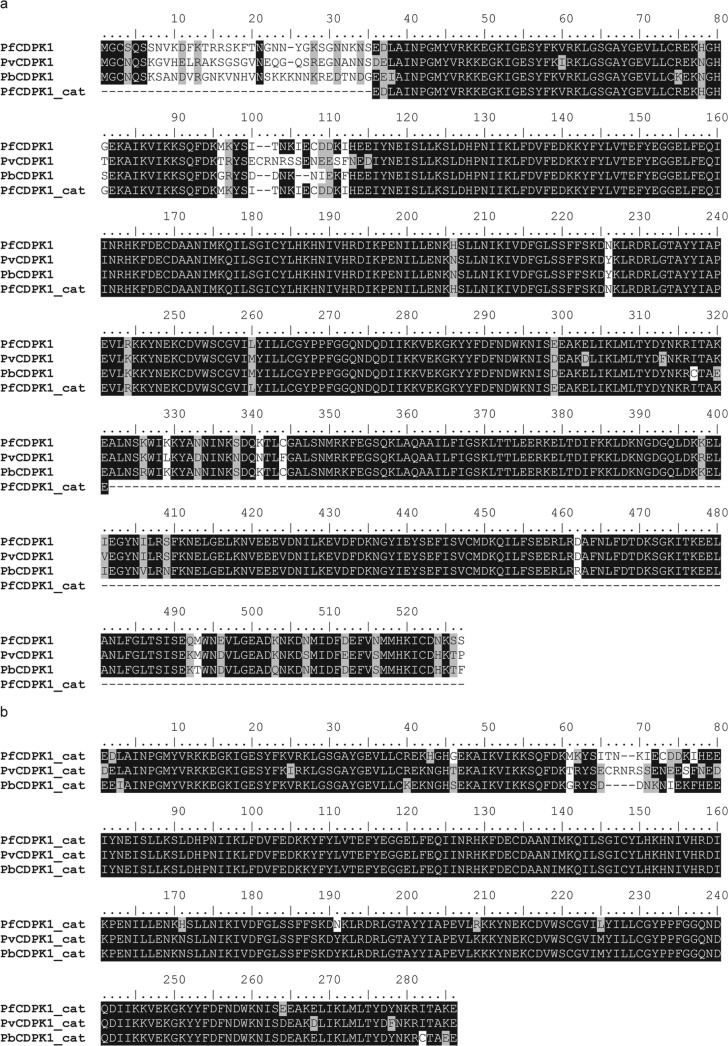
(a and b) Amino acid sequence alignment of full-length CDPK1 proteins (a) and catalytic domains only (b). The overall sequence identities between PfCDPK1 and PbCDPK1 and PvCDPK1 are 88.4% and 87.5%, respectively, and for the catalytic domains, the values are 92.2% and 90.8%, respectively.

Following chemical optimization of the compounds (23, 24), we wanted to monitor inhibition against P. berghei CDPK1 as a prelude to *in vivo* testing in this mouse model of malaria. In addition, we were interested in the inhibitory properties of the compounds against the P. vivax homologue (PvCDPK1), since P. vivax malaria represents a significant threat to health outside Africa for which new treatments are also needed. Therefore, we expressed PbCDPK1 and PvCDPK1 and, using a Rh-ParM ADP biosensor, determined their ATP *K_m_* values to be 160 μM and 30 μM in the absence of MTIP, respectively. Next, we compared the potencies of a panel of 10 PfCDPK1 inhibitors against the activity of all three enzymes (Table 4). Of note, a consideration in the evaluation of potent inhibitors is the so-called tight-binding limit, which is reached when the number of catalytic sites present in the reaction begins to match or exceed the measured IC_50_ of the inhibitor (25). Therefore, for comparative purposes, we considered inhibitors with potencies greater than 10 nM, which is in excess of the enzyme concentration that was used across all three enzymes. Having established the assay conditions, we observed a good correlation between IC_50_s obtained for PfCDPK1 and PbCDPK1 (*r* = 0.96; *P* = 0.00001) or PfCDPK1 and PvCDPK1 (*r* = 0.86; *P* = 0.001). Interestingly, the potency of the compounds for PbCDPK1 was reduced by a factor of 3.6-fold on average (range, 2.5- to 4.8-fold) in comparison to the P. falciparum homologue, while the potency for PvCDK1 was similar or increased slightly compared to PfCDPK1 values (0.8-fold difference; range, 0.5- to 1.0-fold). Considering that both the overall and kinase domain amino acid sequence identity is high between PfCDPK1 and PbCDPK1 (88.4% and 92.2%, respectively) and between PfCDPK1 and PvCDPK1 (87.5% and 90.8%, respectively) (Fig. 6a and b), it was perhaps unsurprising that we found good cross-species inhibition. Nevertheless, we observed a degree of selectivity of our compounds in particular for the P. falciparum and P. vivax CDPK1 enzymes over the P. berghei CDPK1 enzyme, suggesting that there may be subtle differences in the interactions that are made between the inhibitors and the ATP binding pockets between species.

**TABLE 4:** Selectivity of PfCDPK1 inhibitors between Plasmodium species

Compound	IC_50_ (nM)^*a*^			IC_50_ ratio	
	PfCDPK1	PbCDPK1	PvCDPK1	PbCDPK1/PfCDPK1	PvCDPK1/PfCDPK1
27	138 (39.5)	479 (225.4)	68 (16.6)	3.5	0.5
28	40 (19.6)	152 (43.6)	24 (9.2)	3.8	0.6
29	86 (37.8)	392 (193.3)	80 (31.6)	4.6	0.9
40	52 (33.6)	249 (49.4)	48 (26.3)	4.8	0.9
41	73 (19.2)	206 (58.3)	68 (15.5)	2.8	0.9
42	109 (26.4)	350 (56.6)	99 (12.1)	3.2	0.9
43	14 (3.8)	36 (12.2)	12 (1.8)	2.6	0.8
44	107 (25.3)	463 (122.9)	59 (8.1)	4.3	0.6
45	19 (4.1)	58 (4.4)	19 (2.5)	3.0	1.0
46	13 (3.0)	38 (2.4)	11 (2.7)	2.8	0.8

a Mean IC_50_s were determined using ADP detection (Rh-ParM) in the absence of MTIP (*n* = 2) (standard deviations shown in parentheses).

Finally, we have recently reported the *in vivo* antiparasitic properties for a selection of imidazopyrimidazine compounds in the P. berghei mouse model for malaria (26). The biochemical profiles of these compounds are shown in Table 5. All of these compounds were selected as potent inhibitors of PfCDPK1 and PbCDPK1 and were sensitive to the gatekeeper mutation T145Q. Although we were unable to measure IC_50_s below 10 nM with accuracy, it was apparent from the Δ*T_m_* value of 28 K that compound 2 had the strongest affinity for PfCDPK1 and indeed showed the highest potency against P. falciparum growth *in vitro*. However, this compound was inactive *in vivo*, which was attributed to poor plasma exposure consistent with poor absorption properties. With this exception, the remaining examples were found to be effective to various degrees in reducing parasitemia in the P. berghei mouse model. Compounds 6 and 7 displayed the next overall most favorable biochemical profiles including inhibition of PbCDPK1 below 10 nM, combined with EC_50_s against parasite growth below 100 nM. However, these compounds were not significantly more efficacious *in vivo* than compound 1, despite the inferior potency of that compound against PbCDPK1.

**TABLE 5:** *In vitro* biochemical and *in vivo* properties of imidazopyrimidazine inhibitors of PfCDPK1

Compound	Parasite growth inhibition EC_50_ (nM)^*a*^	Δ*T_m_* PfCDPK1 (K)	IC_50_ (nM)^*b*^				% reduction in parasitemia *in vivo* (po)^*c*^	PAMPA *P*_app_ (nm/s)^*d*^	mlogD^*d*^
			WT PfCDPK1	PfCDPK1 T145Q	PbCDPK1	PvCDPK1			
1	439 (216)	16	<10	4,610 (780)	22 (4)	<10	46 (4)	81	3.4
2	13 (1)	28	<10	2,350 (150)	<10	<10	4 (14)	4	0.2
3	407 (32)	21	<10	1,303 (494)	15 (4)	<10	44 (16)	171	3.2
4	297 (154)	22	11 (4)	2,788 (704)	43 (13)	<10	46 (1)	92	2.8
5	294 (167)	20	30 (13)	1,482 (518)	142 (44)	37 (21)	34 (20)	81	3.3
6	77 (32)	22	<10	2,201 (424)	<10	<10	51 (3)	48	3.7
7	75 (34)	23	<10	344 (64)	<10	<10	18 (22)	18	2.2

a Mean EC_50_ values determined by FACS (*n* = 3) (standard deviations [SD] shown in parentheses).

b Mean IC_50_ values determined using Rh-ParM to detect ADP in the absence of MTIP (*n* = 3) (SD shown in parentheses).

c Mean reduction in parasitemia in mice (groups of three mice) given dosage of 50 mg/kg of body weight administered per os (po) (SD in parentheses) (data derived from reference 26).

d Parallel artificial membrane permeability assay (PAMPA) apparent permeability (*P*_app_) (in nm/s) and measured distribution coefficient (mlogD) are *in vitro* ADMET properties (data derived from reference 26).

## DISCUSSION

PfCDPK1 has been identified as a potential therapeutic target for intervention in malaria, and we set out to find novel inhibitors of PfCDPK1 using a high-throughput biochemical assay to screen a library of more than 35,000 small molecules. In the course of developing the HTS assay, we noted an additional substrate-independent ATPase activity, which was absent in the catalytically inactive mutant D191N. The physiological relevance of this activity for PfCDPK1 is unknown; however, ATP hydrolysis by isolated kinases is not uncommon and is considered to be a function of the ATP-binding site, which can be exploited for identification of inhibitors (27). Indeed, we confirmed that hits identified in the ATP depletion assay also inhibited MTIP phosphorylation in a radiometric assay with potencies that were higher in the latter. That observation may have resulted from differences between assay readouts, i.e., the radiometric assay reported kinase activity, while the Kinase-Glo Plus assay reported ATP depletion due to both kinase and ATPase activities. Nevertheless, the Kinase-Glo Plus assay was robust in screening and led to the identification of five chemical series for further characterization. (The structures of all compounds in this study are provided in the supplemental material.) Thermal shift data provided additional evidence that the compounds were binding to PfCDPK1, and thermal stabilization was found to be well correlated with IC_50_s, both within and across chemical series. Such trends have been reported previously, including within a series of diaryl urea inhibitors of p38 mitogen-activated protein (MAP) kinase (28) and across four structural classes of inhibitors of the aspartyl protease BACE (beta site Alzheimer's amyloid precursor protein-cleaving enzyme) (29). Although such a relationship is not always observed, concerning our PfCDPK1 inhibitors it provided a parameter to rank inhibitor affinity beyond the tight-binding limits of the biochemical assay.

By performing IC_50_ determinations in the presence of different ATP concentrations, we found that all five series were competitive with respect to ATP. Series 1 and 2 were selected for chemical optimization based on evident biochemical SARs, good inhibition of parasite growth, and chemical tractability. To further characterize these series, we expressed PfCDPK1 enzymes, replacing a threonine with either a glycine or glutamine residue at the “gatekeeper” position (T145G and T145Q, respectively). Previous studies have shown that kinase ATP site inhibitors may be sensitive to mutation at this position, especially where a small amino acid such as threonine in the case of PfCDPK1 is replaced with one containing a bulkier side chain, while ATP binding is unaffected (22). We found that the affinities of compounds from series 1 and 2 were highly sensitive to the T145Q mutation but not T145G, thus providing further evidence that the compounds were targeted to the ATP-binding site. Subsequently, inhibitor affinity was optimized with reference to a homology model based on the solved crystal structure of Toxoplasma gondii CDPK1 (23, 24). Significant improvements in inhibitor potency against the isolated PfCDPK1 enzyme were achieved leading to examples with biochemical inhibition below 10 nM and Δ*T_m_* values greater than 20 K. These compounds also inhibited the P. vivax homologue of CDPK1 with comparable potencies, suggesting potential for application beyond P. falciparum. However, we did not observe a correlation between inhibition or thermal shift data and parasite growth inhibition. There are a number of possible explanations for this observation, including potential variability in the ability of compounds to cross the red blood cell and/or parasite cell membranes, variations in compound stability over the course of the experiment, sequestration of compounds to a component(s) of the parasite growth medium, and the potential for off-target inhibition of other parasite-encoded kinases. A lack of correlation between inhibitor potency and parasite killing was also reported by Lemercier et al. for a series of imidazopyridazine inhibitors of PfCDPK1 and was suggested to be a limitation, primarily of poor inhibitor membrane permeability (16). To address this, we explored various chemical modifications of series 1 compounds, resulting in improved absorption, distribution, metabolism, excretion, and toxicity (ADMET) properties, including membrane permeability and measured distribution coefficient (mlogD) (24, 26). However, we found that while we could identify compounds with improved membrane permeability combined with potent biochemical inhibition of PfCDPK1, parasite growth inhibition was often reduced, and there was no significant correlation between pIC_50_ and pEC_50_ values irrespective of membrane permeability (parallel artificial membrane permeability assay [PAMPA]) or mlogD. Therefore, although we have shown potent on-target activity and parasite growth inhibition in combination with acceptable ADMET properties, we cannot rule out the possibility that the inhibition observed in the parasite growth assay may not be exclusively attributable to inhibition of this kinase. Nevertheless, profiling against a panel of 73 human kinases indicated that these compounds are not broadly promiscuous (23, 24), and a further investigation into the mechanism of action of these compounds for parasite growth inhibition will be the subject of a future publication.

The lack of correlation between enzymatic inhibition and parasite growth should also be considered in relation to current understanding of the role of PfCDPK1 in the parasite life cycle. Previous studies have shown that PfCDPK1 can be detected throughout the parasite life cycle and that in the asexual stages, it is expressed at the onset of schizogony, with the protein accumulating in the cell until the end of the cycle (8, 13). Although previous attempts to knock out *cdpk1* have been unsuccessful (5, 14), a recent report shows deletion of *Pbcdpk1*. This has brought into question the role of CDPK1 in the late erythrocyte stage of parasite growth, where it was previously believed to be essential (30). Recently, a partial knockdown of PfCDPK1 was achieved by introducing a destabilization domain into the protein without an obvious phenotype (15). However, the authors went on to show that conditional inhibition of PfCDPK1, through overexpression of the kinase-inhibitory J-domain, resulted in an arrest in parasite development in early schizogony, consistent with a role for PfCDPK1 in the late stages of the erythrocytic cycle. As part of a detailed investigation into the role of PbCDPK1 in the sexual stages of the life cycle, Sebastian et al. experimentally exchanged the native *Pbcdpk1* promoter for the weaker *clag* promoter, resulting in undetectable levels of PbCDPK1 in gametocytes and a reduction of at least 10-fold in schizonts (31). Interestingly, there was no effect on the asexual blood stages, suggesting that the bulk of the CDPK1 expressed in the asexual stage is dispensable for survival. Consistent with the latter, we observed only moderate *in vivo* efficacy of our compounds in the P. berghei mouse model, despite good exposure of the compounds in plasma amounting to 35 times the *in vitro* EC_50_ and more than 270 times the PbCDPK1 IC_50_ for compound 6 (23). Taken together, the results from the current study and these recent reports suggest that PfCDPK1 may ultimately be considered dispensable in the blood stages of the parasite life cycle. Nevertheless, targeting the Plasmodium kinome remains an interesting opportunity for antimalarial drug discovery due to the evolutionary divergence from mammalian kinases. Notably, a large-scale cellular phenotypic screen of nearly 2 million compounds for inhibitors of parasite growth in the blood stages resulted in thousands of inhibitors with IC_50_s below 1 μM, a substantial number of which are believed to have the potential to act as kinase inhibitors (32–34). Deconvolution of the relevant pathways and targets from the output of that screen is ongoing, but new targets from within the Plasmodium kinome are beginning to emerge, such as the lipid kinase phosphatidylinositol-4-OH kinase [PI(4)K] (35).

Finally, CDPKs are represented in all members of the Apicomplexa family, including other species responsible for disease in humans and animals such as Toxoplasma gondii, Cryptosporidium parvum, and Eimeria tenella, many of which have high ATP site sequence homologies with PfCDPK1 and small gatekeeper residues (36). Indeed, PfCDPK1 has recently been reported to act as a functional complement for its orthologue in T. gondii (TgCDPK3) by genetic substitution (37). Therefore, the chemical series identified in this study may be suitable for further development against this class of enzymes in related parasitic diseases as well as providing new chemical probes for CDPK1 function in P. falciparum.

## Supplementary Material

Supplemental material
