# Supplementary material for: Biochemical and Antiparasitic Properties of Inhibitors of the Plasmodium falciparum Calcium-Dependent Protein Kinase PfCDPK1
Source: Antimicrob Agents Chemother. 2014 Oct;58(10):6032–43. doi: 10.1128/AAC.02959-14 (PMC4187893; doi:10.1128/AAC.02959-14)
Supplement: Supplemental material [file supp_58_10_6032__index.html]

Biochemical and Antiparasitic Properties of Inhibitors of the Plasmodium falciparum Calcium-Dependent Protein Kinase PfCDPK1 — Supplemental material 

# Biochemical and Antiparasitic Properties of Inhibitors of the Plasmodium falciparum Calcium-Dependent Protein Kinase PfCDPK1

## Supplemental material

**Files in this Data Supplement:**

- Supplemental file 1 -

  Chemical structures (Table S1).

  PDF, 514K
