# Supplementary figures and images for: Biochemical and Antiparasitic Properties of Inhibitors of the Plasmodium falciparum Calcium-Dependent Protein Kinase PfCDPK1
Source: Antimicrob Agents Chemother. 2014 Oct;58(10):6032–43. doi: 10.1128/AAC.02959-14 (PMC4187893; doi:10.1128/AAC.02959-14)

Table S1. Chemical Structures

Compound Series

Structure

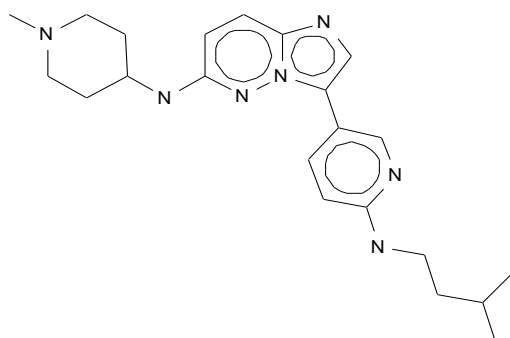

1 1

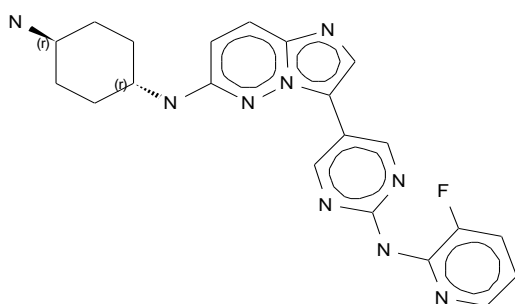

2 1

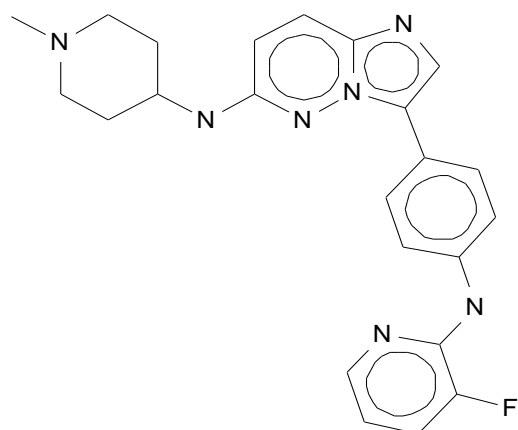

3 1

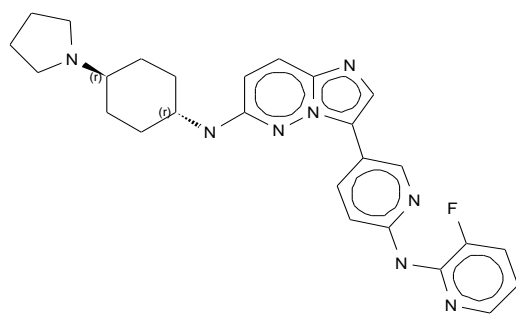

4 1

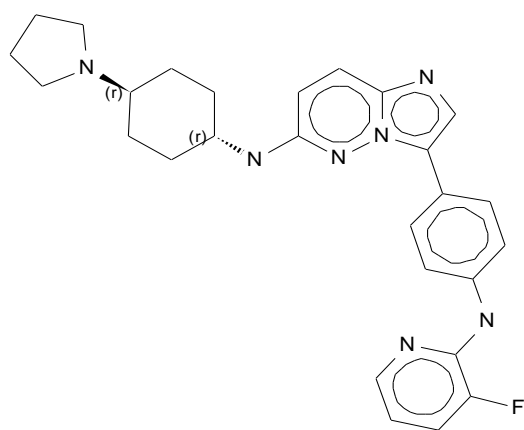

5 1

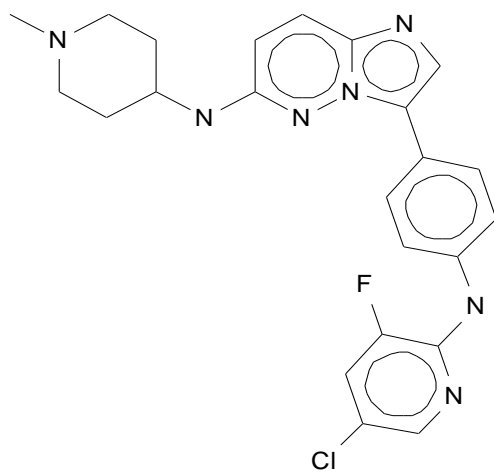

6 1

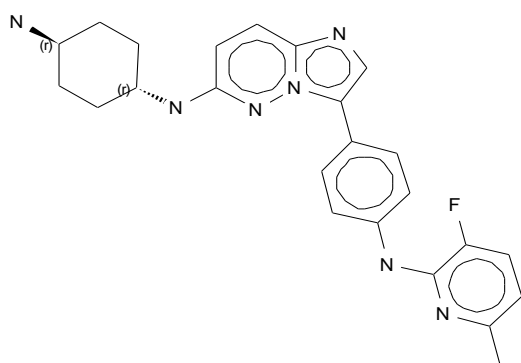

7 1

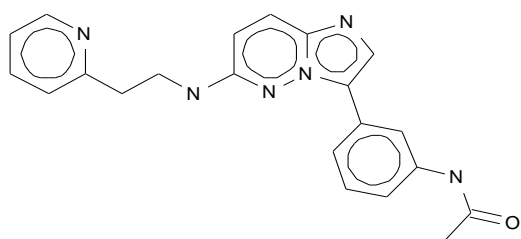

10 1

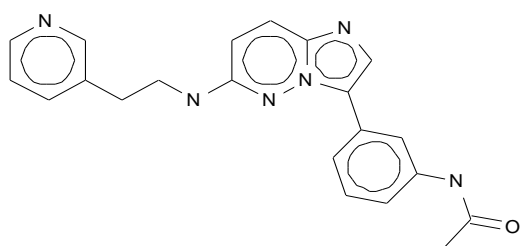

11 1

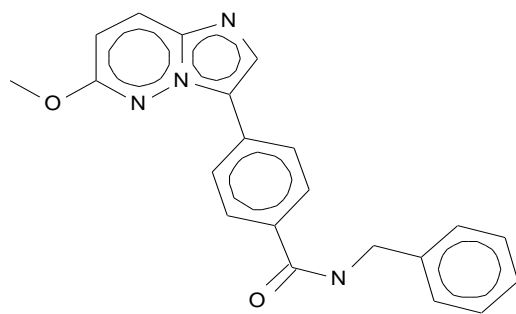

12 1

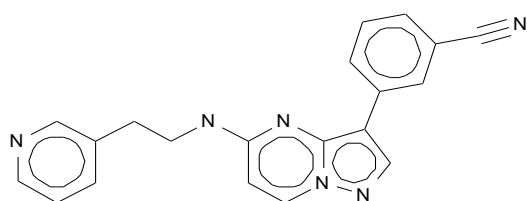

13 2

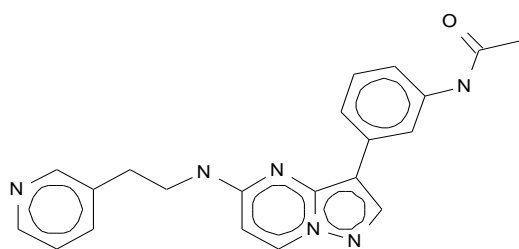

14 2

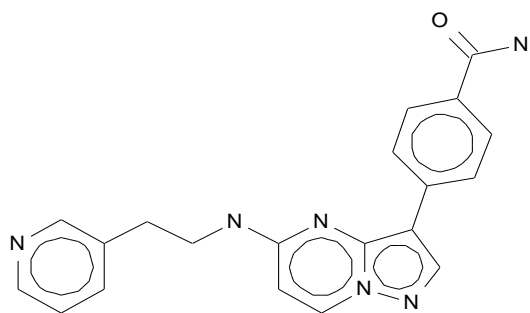

15 2

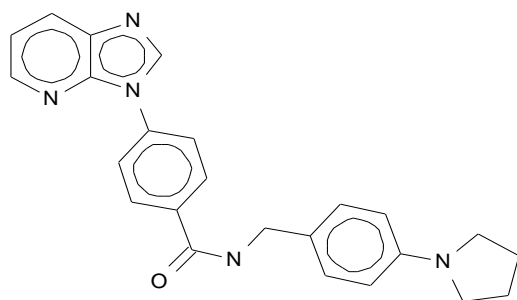

16 3

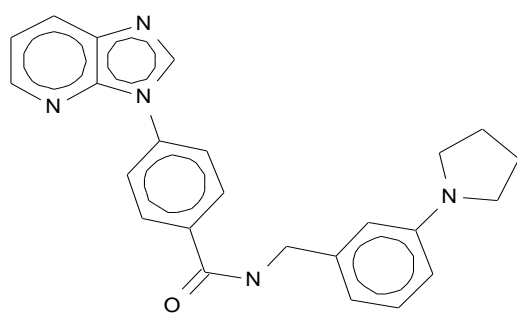

17 3

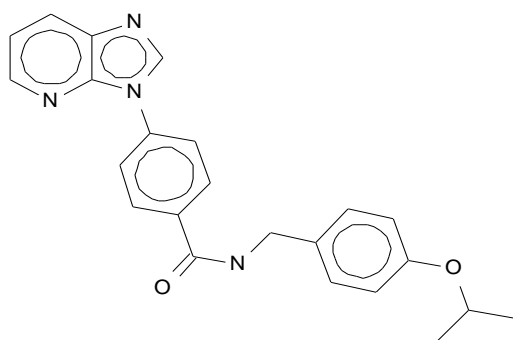

18 3

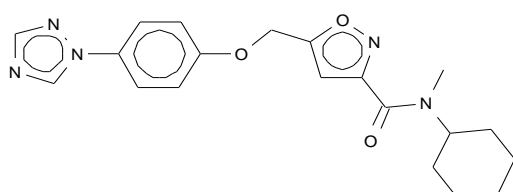

19 4

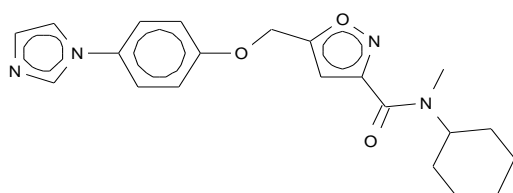

20 4

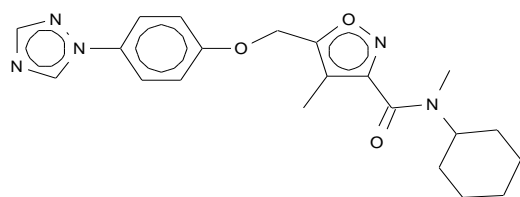

21 4

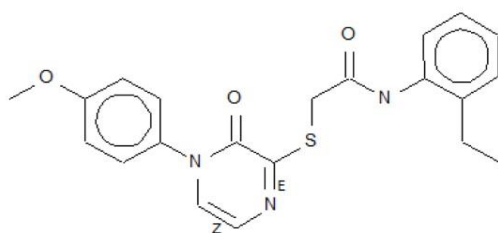

22 5

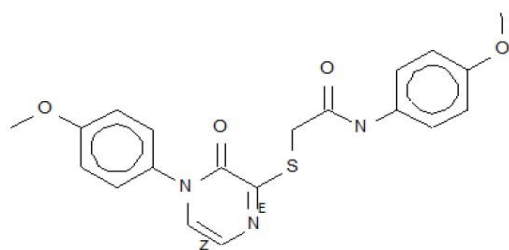

23 5

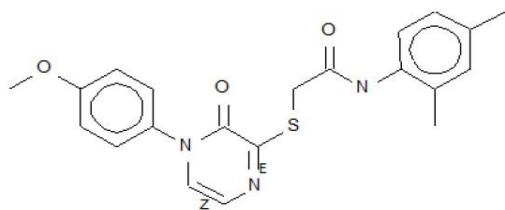

24 5

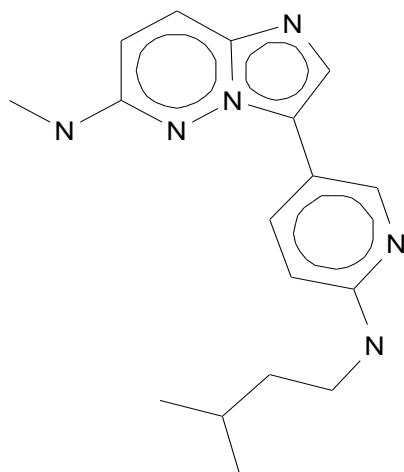

25 1

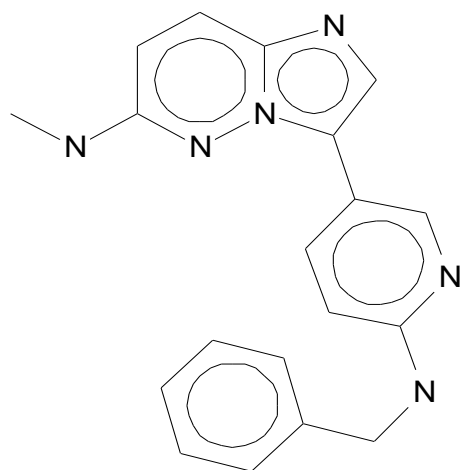

26 1

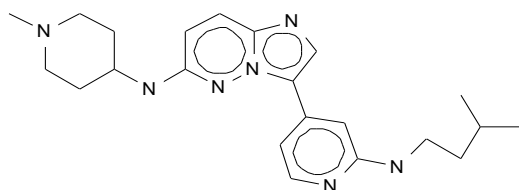

27 1

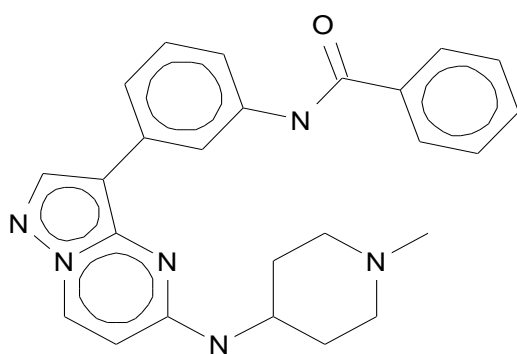

28 2

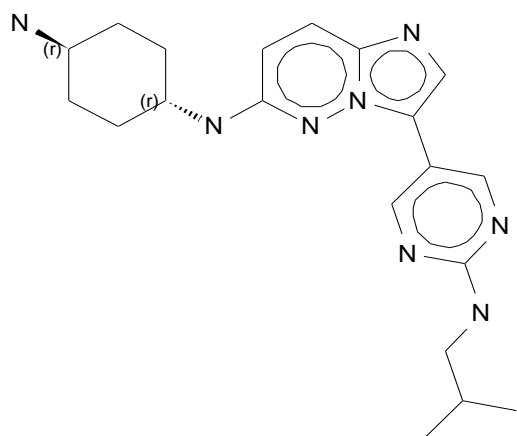

29 1

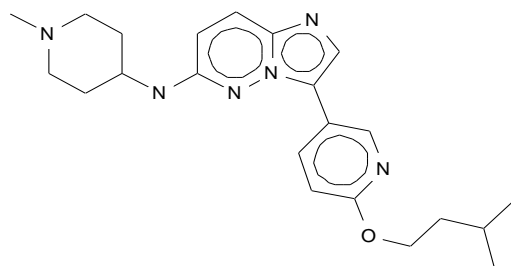

30      1

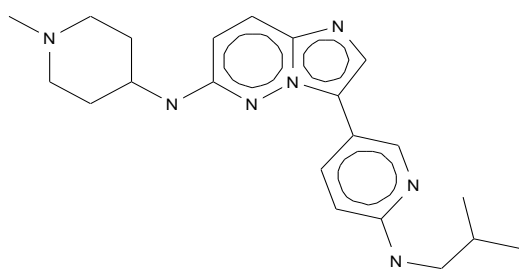

31      1

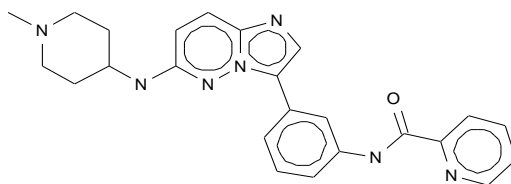

32      1

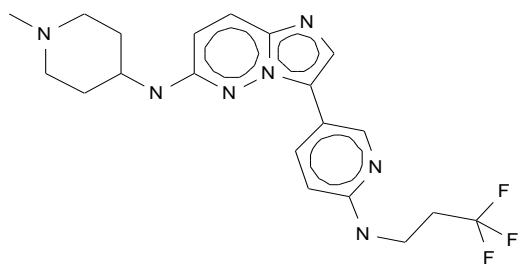

33 1

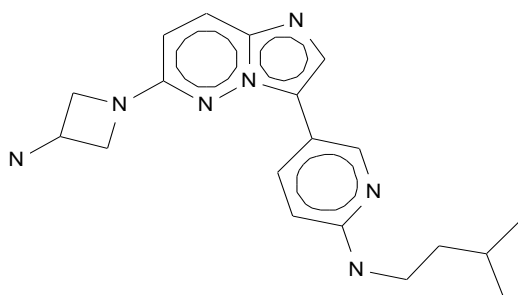

34 1

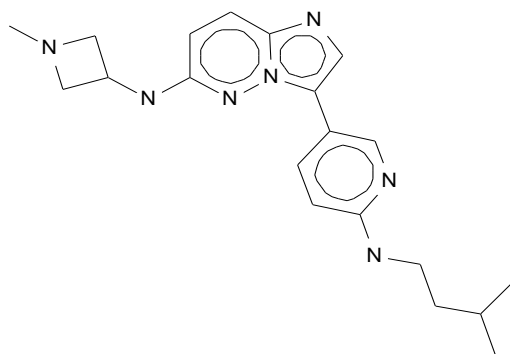

35 1

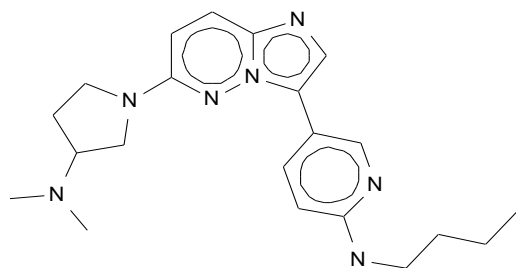

36 1

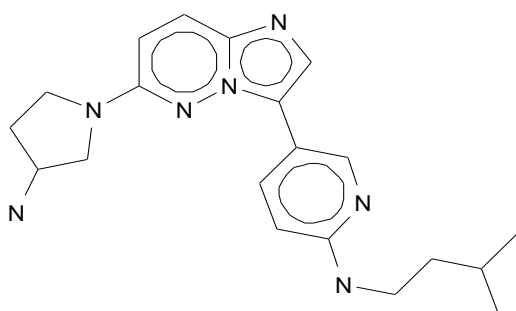

37 1

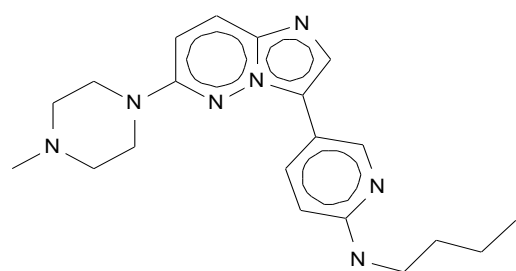

38 1

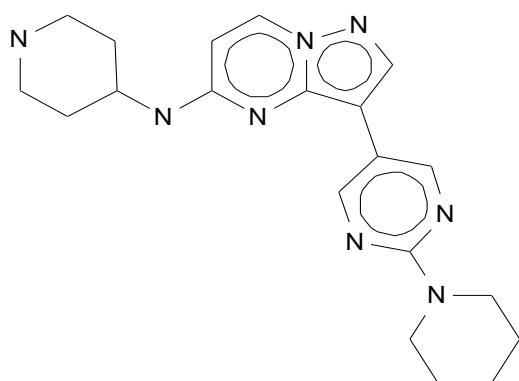

39      2

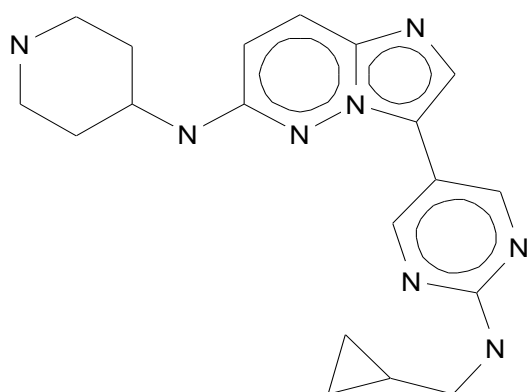

40      1

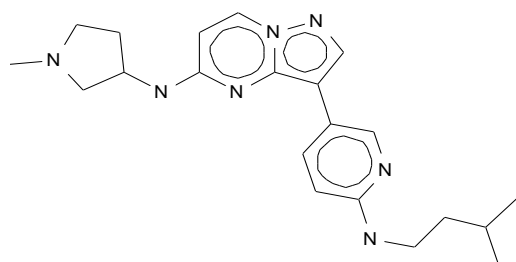

41      2

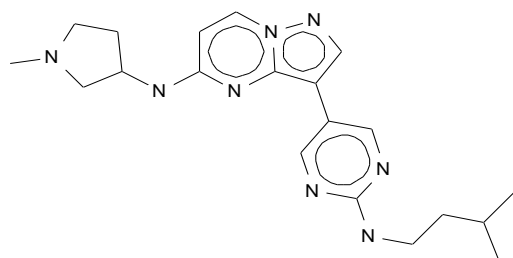

42 2

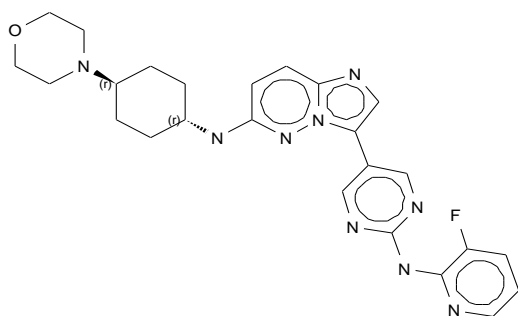

43 1

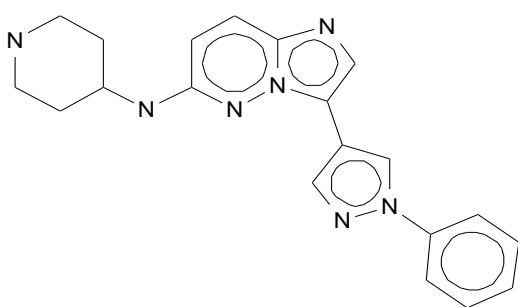

44 1

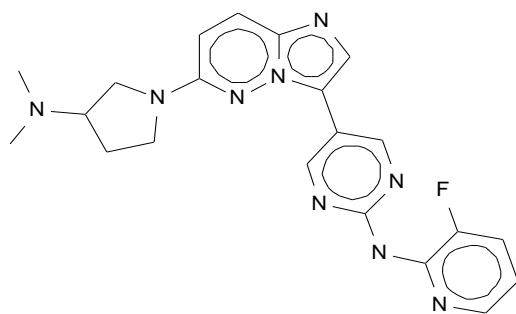

45 1

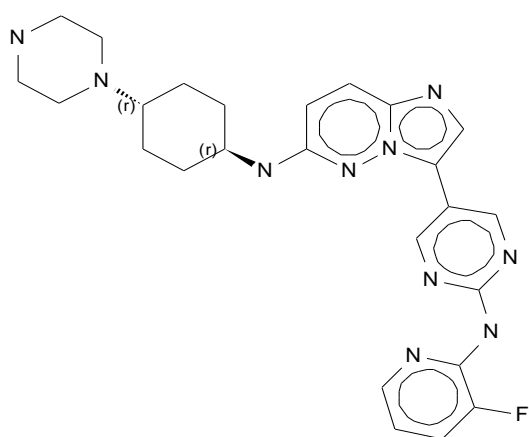

46 1

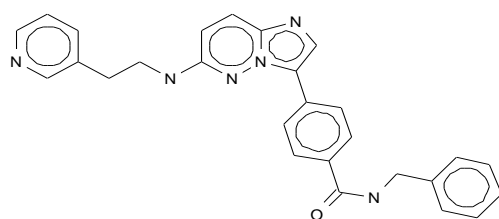

47 1

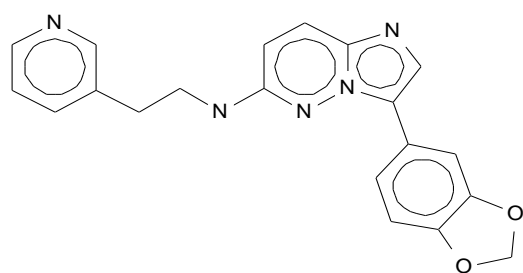

50

1

Supplement: Supplemental material [file AAC.02959-14_zac010143307so1.pdf]
